# The conserved MASRPF motif in the Attractin homolog, Distracted, is required for association with Drosophila E3-ligase Mgrn1

**DOI:** 10.17912/micropub.biology.000416

**Published:** 2021-07-02

**Authors:** Vindhya Nawaratne, Sirisha Kudumala, Priyanka Prakash Kakad, Tanja A Godenschwege

**Affiliations:** 1 Biological Science Department, Florida Atlantic University, Jupiter, FL 33458; 2 Prodigy Biotech, Jupiter, FL 33458; 3 ScienCell Research Laboratories, Inc., 1610 Faraday Avenue, Carlsbad, CA 92008

## Abstract

In rodents, all three paralogs of the Attractin (Atrn) transmembrane protein family exhibit strong phenotypic overlap and are implicated in the regulation of the same G-protein coupled receptors (GPCR) as E3-ligase Mahogunin ring finger 1 (Mgrn1). Recently it was shown that the highly conserved intracellular MASRPF motif in mammal Multiple epidermal growth factor-like domain 8 protein is required for binding of Mgrn1 to mediate ubiquitination of GPCR Smoothened in vitro. Here, we show that the MASRPF motif of *Drosophila* Distracted, the ortholog of ATRN and Attractin-like 1, is required for association with *Drosophila *Mgrn1 (dMgrn1) in vivo.

**Figure 1. In vivo interaction of Distracted and dMgrn1:**
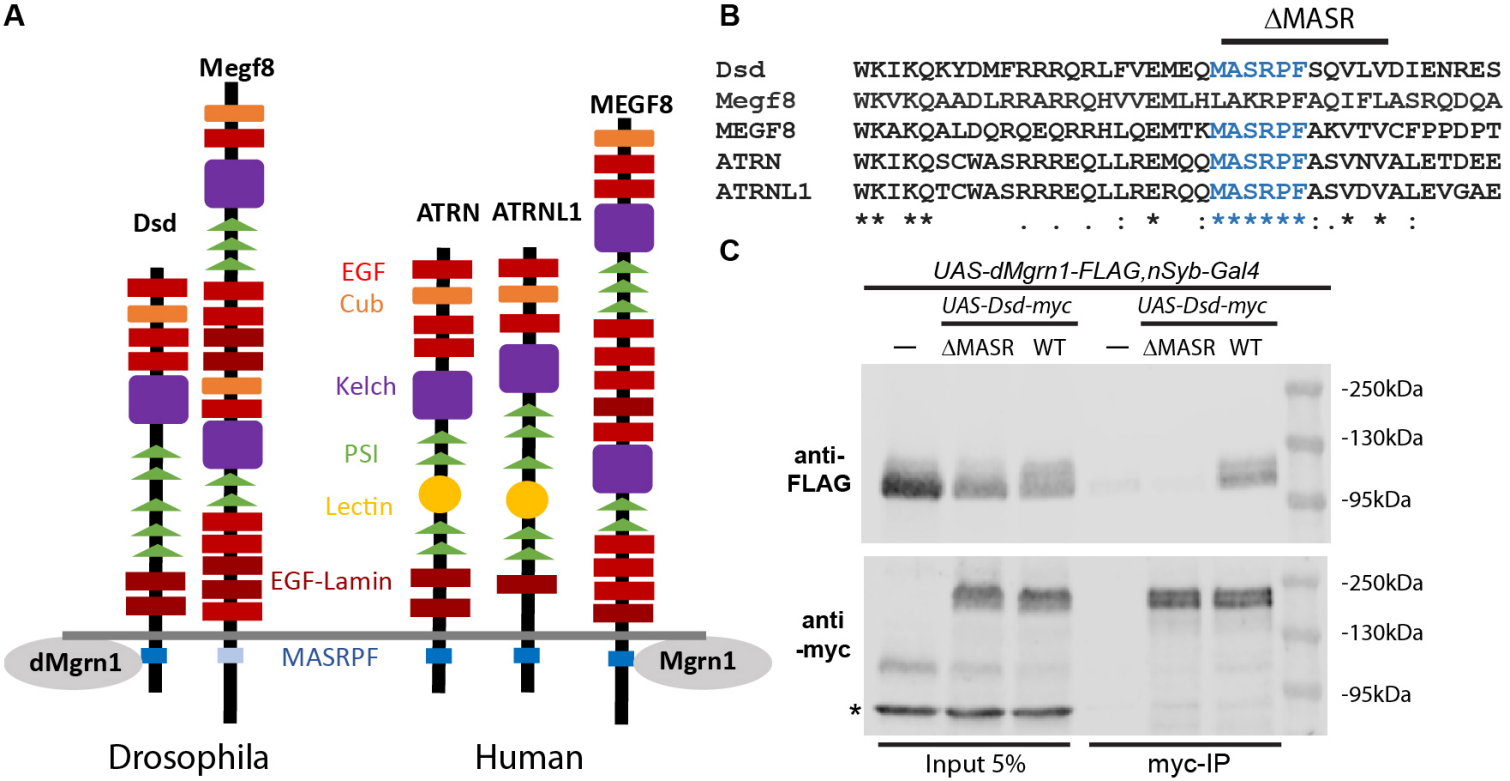
**A.** Comparison of protein motifs of *Drosophila* and human Attractin (ATRN) family orthologs. In mice, the MASRPF motif of Multiple EGF like domains 8 (Megf8) was shown to be essential for Mgrn1 binding (Kong *et al.*, 2020). Here we show that *Drosophila* CG9941 (dMgrn1) associates with Dsd in a MASRPF motif-dependent manner. **B.** Sequence alignment of the first 40 membrane-proximal amino acids in the intracellular domain of Drosophila and human *ATRN* orthologs. In Drosophila, the MASRPF motif (blue) is fully conserved in distracted(*dsd*), but only partially in the *Megf8* gene. Bar indicates amino acids deleted in the myc-tagged *Dsd*Δ*MASR* construct. **C.** Co-immunoprecipitation of Dsd and dMgrn1. Top blot was probed with anti-FLAG antibodies for dMgrn1, and subsequently probed with anti-myc antibodies for Dsd-myc IP (bottom blot). First three lanes show 5% of the input of protein homogenates obtained from Drosophila heads that were panneuronally (*nSyb-Gal4* driver) expressing FLAG-tagged *UAS-dMgrn1* (lane 1), co-expressing *UAS-dMgrn1-FLAG* with *UAS-Dsd*Δ*MASR-myc* (lane 2) or *UAS-Dsd-myc* (lane 3). Lanes 3-6 show immunoprecipitation (IP) for myc-tagged proteins of the supernatant containing only dMgrn1-FLAG (lane 4, negative control), dMgrn1-FLAG and DsdΔMASR-myc (lane 5), or dMgrn1-FLAG and wildtype (WT) Dsd-myc (lane 6). dMgrn1-FLAG is detected at approximately 110kDa and Dsd-myc at 160kDa. Asterisk indicates an unspecific protein detected by the antibodies.

## Description

In vertebrates, the Attractin (Atrn) transmembrane protein family is comprised of ATRN, Attractin-like 1 (ATRNL1) and Multiple epidermal growth factor-like domain 8 (MEGF8) proteins, which contain an extensive extracellular domain with numerous protein interaction motifs and a highly conserved intracellular MASRPF motif (Fig. 1A and B) (Walker *et al.*, 2007). Common to all three paralogs is that they have a strong phenotypic overlap with E3-ligase Mahogunin ring finger 1 (Mgrn1), due to regulation of the same G-protein coupled receptors (GPCRs) as Mgrn1. For example, rodent Atrn and Mgrn1 negatively regulate signaling melanocortin receptors MCR1 and MCR4 by promoting their endolysosomal trafficking. Thus, loss of function mutations in *Atrn* or *Mgrn1* suppress the pigmentation and obesity phenotypes of MCR1/4 antagonist gain of function mutants (Barsh *et al.*, 2002; He *et al.*, 2003; Nagle *et al.*, 1999). Loss of function of *Megf8* and *Mgrn1* mutants exhibit common developmental phenotypes (Aune *et al.*, 2008; Cota *et al.*, 2006; Zhang *et al.*, 2009) and both proteins were identified as negative regulators of the GPCR Smoothened in mice (Pusapati *et al.*, 2018). Most recently it was shown in vitro that Mgrn1 and Megf8 co-immunoprecipitate in a MASRPF motif-dependent manner, and this association is required for Mgrn1 to mediate membrane-tethered ubiquitination to modulate the signaling strength of Hedgehog morphogens in mice (Kong *et al.*, 2020).

Distracted (*dsd*) is the sole *Drosophila* ortholog of human ATRN and ATRNL1 (Fig. 1A), while *Drosophila* Megf8*,* like rodent Megf8, is required for early development (Lloyd *et al.*, 2018). In contrast to *Drosophila* Megf8, the MASRPF motif is fully conserved in Dsd (Fig. 1B). The DRSC Integrative Ortholog Prediction Tool uses 15 different algorithms to identify orthologous genes (https://www.flyrnai.org/cgi-bin/DRSC_orthologs.pl, Version 8.0, August 2019). For *CG9941* (hereafter referred to as *dMrgn1*) more algorithms predicted that it is orthologous to mammal *MGRN1 (human 11/15, rat 7/13, mouse 10/15)* than for *RNF157* (Ring Finger Protein 157; human 10/14, rat 4/15, mouse 10/15). While the zinc finger domain is highly conserved (83% amino acid identity) in dMgrn1 when compared to either mammal MGRN1 or RNF157 proteins, the entire protein is less well preserved (amino acid identity 35-37%/similarity 48-51%) in part due to the *Drosophila* protein being larger by approximately 210 amino acids when compared to mammalian Mgrn1.

Here, we generated myc-tagged Dsd containing (*UAS-Dsd-myc*) or lacking the MASRPF motif (*UAS-Dsd*Δ*MASR-myc*, Fig. 1B), and FLAG-tagged dMgrn1 (*UAS-dMgrn1-FLAG*) transgenic lines to determine whether Dsd and dMgrn1 are in a complex together in vivo, and if the interaction is dependent on the MASRPF motif. We panneuronally (*nSyb-Gal4*) expressed dMgrn1-FLAG alone or together with Dsd-myc or DsdΔMASR-myc, and used Drosophila heads to perform immunoprecipitation (IP) with myc-trap agarose beads. In western blots of fly head supernatant prior to IP, dMgrn1-FLAG is detected at 110 kDa and Dsd-myc at 160kDa (Fig. 1, lane 1-3) confirming protein expression. Multiple independent IP experiments were performed using two distinct transgenic lines for expression of Dsd-myc and DsdΔMASR-myc, while using the same transgenic line for expression of dMgrn1-FLAG. The results demonstrated that Dsd and dMgrn1 co-IP, suggesting they interact in vivo (n=4), and that this interaction was indeed dependent on the MASRPF motif (n=3, Fig. 1C). After co-IP, dMgrn1-FLAG protein was detected abundantly when it was co-expressed with Dsd-myc (Fig. 1C, lane 6) but absent or at similarly low levels as the negative controls (*UAS-dMgrn1-FLAG,nSyb-Gal4,* Fig. 1C, lane 4) when co-expressed with DsdΔMASR-myc (Fig. 1C, lane 5). These results imply that Drosophila is a valid model to study ATRN’s proposed role as a substrate adaptor for MGRN1 (Kong *et al.*, 2020). Loss of function of Atrn and Mgrn1 in rodents is associated with mitochondrial dysfunction, elevated oxidative stress, and adult-onset spongiform neurodegeneration (Bronson *et al.*, 2001; Cota *et al.*, 2008; He *et al.*, 2003; Kuramoto *et al.*, 2001; Li *et al.*, 2014; Muto and Sato, 2003; Paz *et al.*, 2007). In humans, ATRN is a candidate gene for sporadic amyotrophic lateral sclerosis (Morahan *et al.*, 2009), ATRN plasma levels are altered in early-onset Alzheimer’s disease (AD) patients prior to symptoms (Muenchhoff *et al.*, 2016), and its gene expression is reduced in Parkinson disease (PD) patients (Glaab and Schneider, 2015)**.** Since theATRN/MGRN1 receptor substrates involved in nervous system survival have not been identified, future studies in Drosophila may provide insight into the relevant signaling pathways.

## Methods

*Cloning and mutagenesis of Dsd and dMgrn1 transgenes*

All primers were generated by Integrated DNA Technologies (IDT, Iowa, USA). The Dsd open reading frame (ORF) without the stop codons was amplified with forward (CAC CAT GTC CCT GTT GCC ACC G) and reverse (TGT ACA ACT GTC TGG GTG CTG GCT CTG) primers from the LD14047 cDNA clone (Stock Number: 1301485, Drosophila Genomics Resource Center, Indiana, USA) and cloned into the pENTR/D-TOPO (Invitrogen, Massachusetts, USA). The dMgrn1 open reading frame without the stop codons was amplified with forward (ATG GGC AAC TTG TGA GCA G) and reverse (AAC ATT GAC GGC ATT CTG) primers from the LP12254 cDNA clone (Stock Number: 1344980, Drosophila Genomics Resource Center, Indiana, USA) and cloned into the pENTR/D-TOPO . In addition, the highly conserved motif MASRPFSQVLV (amino acids 1208-1218) was deleted in pENTR/D-TOPO-DSD entry clone with QuikChange Lightning Site-Directed Mutagenesis Kit (Agilent, California, USA) using forward (CGT CGA AAT GGA ACA GGA TAT CGA GAA CCG CG) and reverse (CGC GGT TCT CGA TAT CCT GTT CCA TTT CGA CG) primers. The vectors pENTR/D-TOPO containing *Mgrn1*, *Dsd* without or with the MASRPFSQVLV deletion (referred to as DsdΔMASR) were verified using restriction enzyme digests and Sanger sequencing (Genewiz, New Jersey, USA). Both *Dsd* entry vectors were recombined with the pTWM destination vector (Stock Number: 1107, Drosophila Genomics Resource Center, Indiana, USA) and the *dMgrn1* entry vector with the pTWF destination vector (Stock Number: 1116, Drosophila Genomics Resource Center, Indiana, USA) using LR clonase. The recombination for the presence of six in-frame myc-codons for the *Dsd* constructs and the three in-frame FLAG codons for the Mgrn1 construct at the C-termini was verified using restriction enzyme digests and Sanger sequencing (Genewiz, New Jersey, USA). The multiple UAS-transgenic lines with P-element insertions at random sites on the second and third chromosome for all three constructs were established in the *w^1118^* background by BestGene Inc (California, USA).

*Fly strains and maintenance*

The *nSyb-Gal4* line (Bloomington Stock Center, Indiana, USA, RRID: BDSC_51635) was recombined with the *UAS-dMgrn1-FLAG* line to established a stable stock expressing dMgrn1-FLAG panneuronally. The *UAS-dMgrn1-FLAG,nSyb-Gal4* stock was crossed to two distinct *UAS-Dsd-myc* or the *UAS-Dsd*Δ*MASR-myc* lines and the offspring were collected for the co-IP experiments. The *UAS-dMgrn1-FLAG,nSyb-Gal4* stock was used as a negative control. All flies were reared on standard fly media at 25°C.

*Co- immunoprecipitation*

Collected adult flies (~3ml) were snap-frozen in liquid nitrogen in 15ml conical tubes, heads were severed by vigorous shaking, and collected by passing through a sieve. Samples were kept at 4^o^C for the rest of the experiment. 100mg of heads were homogenized in 450μl of lysis buffer (10 mM Tris base, 150 mM NaCl, 0.5 mM EDTA, 0.5% NP-40, 10µM MG132, 1 mM PMSF, 20mM NEM, 1x Complete ULTRA EDTA-free Protease Inhibitor cocktail, 1xPhosStop) using a handheld homogenizer. Homogenates were centrifuged at 500xg for 1min to remove cuticle particulates, and the supernatant was collected and centrifuged again at 16000xg for 30min. 5% of the supernatant was saved as inputs while the rest of the supernatant was precleared with 60μl of Protein A agarose beads (Pierce™ Protein A Agarose, ThermoFisher Scientific, Massachusetts, USA) for 10min. Protein A agarose beads were removed by centrifugation at 2500xg for 2 min and supernatant immunoprecipitated with 25μl of Myc-Trap® (ChromoTek, Bavaria, Germany) agarose beads for 1hr. Beads were collected by centrifugation at 2500xg for 2 min, washed 3 times with 500μldilution buffer (10mM Tris base, 150mM NaCl), resuspended in 30μl 2x Laemmli buffer, boiled for 10 min at 95°C and pelleted at 2500xg for 2 min. Proteins in the supernatant were separated on an 8% SDS-PAGE gel. Western blots of the gels were first probed with mouse anti-FLAG^®^ M2 (Sigma-Aldrich, Missouri, USA, RRID:AB_259529, dilution 1:1000) and with mouse anti-myc 9E11 antibody (BioLegend, California, USA, RRID: AB_2565039, dilution 1:1000) consecutively. Goat anti-mouse IRDye® 680RD (LI-COR Biosciences, Nebraska, USA, RRID:AB_10956588, dilution 1:15000) was used to detect the primary antibodies. Images were acquired and analyzed using Image Studio from LI-COR Biosciences.

## References

[R1] Alberts MJ (2001). Suppression of recurrent transient ischemic attacks by a statin agent.. Neurology.

[R2] Aune CN, Chatterjee B, Zhao XQ, Francis R, Bracero L, Yu Q, Rosenthal J, Leatherbury L, Lo CW (2008). Mouse model of heterotaxy with single ventricle spectrum of cardiac anomalies.. Pediatr Res.

[R3] Barsh GS, He L, Gunn TM (1970). Genetic and biochemical studies of the Agouti-attractin system.. J Recept Signal Transduct Res.

[R4] Cota CD, Bagher P, Pelc P, Smith CO, Bodner CR, Gunn TM (2006). Mice with mutations in Mahogunin ring finger-1 (Mgrn1) exhibit abnormal patterning of the left-right axis.. Dev Dyn.

[R5] Cota CD, Liu RR, Sumberac TM, Jung S, Vencato D, Millet YH, Gunn TM (2008). Genetic and phenotypic studies of the dark-like mutant mouse.. Genesis.

[R6] Glaab E, Schneider R (2014). Comparative pathway and network analysis of brain transcriptome changes during adult aging and in Parkinson's disease.. Neurobiol Dis.

[R7] He L, Eldridge AG, Jackson PK, Gunn TM, Barsh GS (2003). Accessory proteins for melanocortin signaling: attractin and mahogunin.. Ann N Y Acad Sci.

[R8] He L, Lu XY, Jolly AF, Eldridge AG, Watson SJ, Jackson PK, Barsh GS, Gunn TM (2003). Spongiform degeneration in mahoganoid mutant mice.. Science.

[R9] Kong JH, Young CB, Pusapati GV, Patel CB, Ho S, Krishnan A, Lin JI, Devine W, Moreau de Bellaing A, Athni TS, Aravind L, Gunn TM, Lo CW, Rohatgi R (2020). A Membrane-Tethered Ubiquitination Pathway Regulates Hedgehog Signaling and Heart Development.. Dev Cell.

[R10] Kuramoto T, Kitada K, Inui T, Sasaki Y, Ito K, Hase T, Kawagachi S, Ogawa Y, Nakao K, Barsh GS, Nagao M, Ushijima T, Serikawa T (2001). Attractin/mahogany/zitter plays a critical role in myelination of the central nervous system.. Proc Natl Acad Sci U S A.

[R11] Li J, Yang J, Cheng D, Shen SL, Xiong CL (2013). New clues to identify proteins correlated with Attractin.. Andrologia.

[R12] Lloyd DL, Toegel M, Fulga TA, Wilkie AOM (2018). The Drosophila homologue of MEGF8 is essential for early development.. Sci Rep.

[R13] Morahan JM, Yu B, Trent RJ, Pamphlett R (1970). A genome-wide analysis of brain DNA methylation identifies new candidate genes for sporadic amyotrophic lateral sclerosis.. Amyotroph Lateral Scler.

[R14] Muenchhoff J, Poljak A, Thalamuthu A, Gupta VB, Chatterjee P, Raftery M, Masters CL, Morris JC, Bateman RJ, Fagan AM, Martins RN, Sachdev PS (2016). Changes in the plasma proteome at asymptomatic and symptomatic stages of autosomal dominant Alzheimer's disease.. Sci Rep.

[R15] Muto Y, Sato K (2003). Pivotal role of attractin in cell survival under oxidative stress in the zitter rat brain with genetic spongiform encephalopathy.. Brain Res Mol Brain Res.

[R16] Nagle DL, McGrail SH, Vitale J, Woolf EA, Dussault BJ Jr, DiRocco L, Holmgren L, Montagno J, Bork P, Huszar D, Fairchild-Huntress V, Ge P, Keilty J, Ebeling C, Baldini L, Gilchrist J, Burn P, Carlson GA, Moore KJ (1999). The mahogany protein is a receptor involved in suppression of obesity.. Nature.

[R17] Paz J, Yao H, Lim HS, Lu XY, Zhang W (2006). The neuroprotective role of attractin in neurodegeneration.. Neurobiol Aging.

[R18] Pusapati GV, Kong JH, Patel BB, Krishnan A, Sagner A, Kinnebrew M, Briscoe J, Aravind L, Rohatgi R (2018). CRISPR Screens Uncover Genes that Regulate Target Cell Sensitivity to the Morphogen Sonic Hedgehog.. Dev Cell.

[R19] Walker WP, Aradhya S, Hu CL, Shen S, Zhang W, Azarani A, Lu X, Barsh GS, Gunn TM (2007). Genetic analysis of attractin homologs.. Genesis.

[R20] Zhang Z, Alpert D, Francis R, Chatterjee B, Yu Q, Tansey T, Sabol SL, Cui C, Bai Y, Koriabine M, Yoshinaga Y, Cheng JF, Chen F, Martin J, Schackwitz W, Gunn TM, Kramer KL, De Jong PJ, Pennacchio LA, Lo CW (2009). Massively parallel sequencing identifies the gene Megf8 with ENU-induced mutation causing heterotaxy.. Proc Natl Acad Sci U S A.

